# Financial Incentives Differentially Regulate Neural Processing of Positive and Negative Emotions during Value-Based Decision-Making

**DOI:** 10.3389/fnhum.2018.00058

**Published:** 2018-02-13

**Authors:** Anne M. Farrell, Joshua O. S. Goh, Brian J. White

**Affiliations:** ^1^Department of Accountancy, Farmer School of Business, Miami University, Oxford, OH, United States; ^2^Graduate Institute of Brain and Mind Sciences, College of Medicine, National Taiwan University, Taipei, Taiwan; ^3^Department of Psychology, National Taiwan University, Taipei, Taiwan; ^4^Neurobiology and Cognitive Science Center, National Taiwan University, Taipei, Taiwan; ^5^Center for Artificial Intelligence and Advanced Robotics, National Taiwan University, Taipei, Taiwan; ^6^Department of Accounting, McCombs School of Business, The University of Texas at Austin, Austin, TX, United States

**Keywords:** positive and negative emotion, emotion regulation, financial incentives, decision-making, fMRI

## Abstract

Emotional and economic incentives often conflict in decision environments. To make economically desirable decisions then, deliberative neural processes must be engaged to regulate automatic emotional reactions. In this functional magnetic resonance imaging (fMRI) study, we evaluated how fixed wage (FW) incentives and performance-based (PB) financial incentives, in which pay is proportional to outcome, differentially regulate positive and negative emotional reactions to hypothetical colleagues that conflicted with the economics of available alternatives. Neural activity from FW to PB incentive contexts decreased for positive emotional stimuli but increased for negative stimuli in middle temporal, insula, and medial prefrontal regions. In addition, PB incentives further induced greater responses to negative than positive emotional decisions in the frontal and anterior cingulate regions involved in emotion regulation. Greater response to positive than negative emotional features in these regions also correlated with lower frequencies of economically desirable choices. Our findings suggest that whereas positive emotion regulation involves a reduction of responses in valence representation regions, negative emotion regulation additionally engages brain regions for deliberative processing and signaling of incongruous events.

## Introduction

When emotional features conflict with financial features of value-based decisions, individuals tend to forego financially desirable choices and make choices that reflect emotional reactions instead (Kida et al., 2001; Moreno et al., 2002; Thaler et al., 2013). Such affective biases are not immutable and appropriate incentives motivate individuals towards more objective decisions despite conflicting emotional contexts (Cohen, 2005; Phelps et al., 2014). Critically, positive and negative emotional reactions involve distinct neural processes (Vytal and Hamann, 2010). Yet how differential emotional valences operate on objective incentive processing in decisions remains unclear. In this study, we applied the common example of corporations compensating managers with fixed wages (FWs) that are independent of investment choices or with performance-based (PB) financial incentives proportional to economic payoffs from investments. Using this approach, we evaluated how monetary incentives modulate the neural processing of positive and negative emotional reactions to colleagues when individuals make value-based decisions about investment proposals.

Studies have shown that neural activity in medial frontal, medial parietal, temporoparietal junction, insula and medial temporal areas track emotional valence of stimuli in a relatively automatic and reactive manner (Epstein, 1994; LeDoux, 2000; Cohen, 2005; Evans, 2006, 2008; Phelps et al., 2014). By contrast, the regulation of emotions when monetary payoffs conflict requires more deliberative processing involving lateral frontal activity (Berkman and Lieberman, 2009; Ochsner et al., 2012). In support of this, we previously reported higher neural activity during emotion-laden than emotion-neutral fixed-wage decisions in the superior medial frontal/anterior cingulate, posterior cingulate/precuneus, bilateral inferior temporal and left insula regions (Farrell et al., 2014). Critically, PB incentives additionally evoked greater activity than FWs during emotion-laden decisions in bilateral middle temporal, frontal, and striatal areas that were associated with more economical desirable decisions. Here, we further examined previous data and considered that FW and PB financial incentives should also have distinct effects on conflicting positive and negative emotional reactions during value-based decision processing.

In a FW context there is no personal economic cost to individuals in making choices consistent with subjective emotions rather than with monetary payoffs to the organization. Investment choices associated with positive emotions compared to negative emotions should be more valuable to the individual, for instance in proposals by favorable vs. antagonistic colleagues, albeit emotionally but not economically. Consequently, we expected that neural responses in a FW context should be higher in positive than in negative emotion contexts in brain regions sensitive to emotional valence.

PB financial incentives, however, are linked to choice outcomes so that additional neural processing is needed to choose economically desirable options that are now personally relevant and that might conflict with emotional reactions. Specifically, *rejecting* economically subpar investments associated with positive emotions should require cognitive effort to ignore automatic neural responses to positive affective stimuli. Also, *accepting* economically desirable investments associated with negative emotions should require cognitive effort to enhance processing of the economic value of the choice as well as to regulate aversion to the negative affective stimuli. We considered that this latter set of operations to handle negative affect might involve double penalty such that greater neural resources are required compared to operations on positive stimuli (Ochsner et al., 2012). Consequently, neural responses should be higher to conflicting negative emotions than in positive emotion contexts in brain regions processing emotional valence in accordance with the goal to prioritize the now-relevant performance incentives. In addition, we were interested in whether higher neural activity in lateral frontal areas would also be engaged for negative than positive emotion contexts reflecting greater need for regulating negative than positive emotional reactions (Berkman and Lieberman, 2009; Ochsner et al., 2012).

## Materials and Methods

### Participants

Participants were 27 right-handed, male, native English-speaking graduate business students. We restricted our scope to male participants, the majority of our recruitment sample pool, to focus on neural activity associated with emotional and incentive contexts. We note that sex differences are possible but would require further investigation in future studies. Analyses of behavioral performance were based on data from all participants. Three participants moved excessively during the functional magnetic resonance imaging (fMRI) experiment (more than 3 mm or 3° based on one voxel size; see brain imaging protocol below) and were excluded from the brain image analysis, which was thus based on the remaining 24 participants. Participants were remunerated for their work performance based on the investment choice task fMRI experiment. On average, participants earned $45.87 (SD = $1.00; range: $45.00–$47.00). This study was carried out in accordance with the recommendations of the institutional review board at the University of Illinois at Urbana-Champaign with written informed consent from all subjects. All subjects gave written informed consent in accordance with the Declaration of Helsinki. The protocol was approved by the institutional review board at the University of Illinois at Urbana-Champaign.

### Investment Choice Task Stimuli

To create the stimuli for the investment choice task, we used 21 black-and-white portrait photos of middle-aged white males in business suits with smiling or neutral facial expressions to portray hypothetical division managers. Photographs were selected from a larger set based on the efficacy of inducing positive, negative and neutral affect in a separate sample of 13 male raters and the specific faces applied to hypothetical situations accordingly below (see Farrell et al., 2014 for more details). Six of the photos were used to portray familiar managers and were used in the instructional materials presented to participants prior to entering the scanner (Supplementary Text 1). These six photos were associated with text descriptions of hypothetical prior interactions the participant had had with each of the managers. Of the six descriptions, two were designed to induce positive emotional reactions to the hypothetical managers (hereafter, positive emotion managers), two to induce negative emotional reactions (negative emotion managers), and two to induce no emotional reactions (neutral emotion managers). The remaining 15 photos all had neutral facial expressions to avoid any emotional reactions, were not associated with descriptions of hypothetical prior interactions, and were not used in the pre-scanner instructional materials (hereafter, unfamiliar managers). We paired each of the six familiar managers with each of the 15 unfamiliar managers to create 90 different choice pairings, instantiating three conditions used in the fMRI experiment: 30 choices with positive emotion managers and unfamiliar managers (POS), 30 with negative emotion managers and unfamiliar managers (NEG), and 30 with neutral emotion managers and unfamiliar managers (NEU).

**Figure 1:**
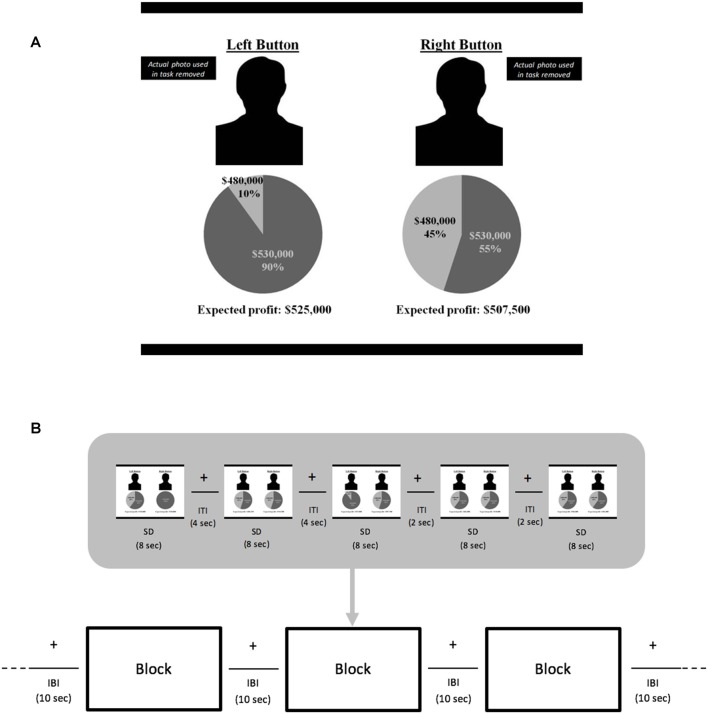
Blocked-design functional magnetic resonance imaging (fMRI) experiment. Samples of a **(A)** negative stimulus from **(B)** trials comprising a block. Manager facial photographs are not shown due to copyright issues. Left button photo depicts a manager showing negative emotional facial expression and the right button photo a manager with neutral expression. For each pay type condition, fixed wage (FW) and performance-based (PB), there were three functional runs. In each run, 90 investment choice stimuli trials were randomly distributed so that there were 30 stimuli per run. Each run had six blocks of five stimuli each—two blocks pairing positive emotion and unfamiliar managers, two with negative emotion and unfamiliar managers, and two with neutral emotion and unfamiliar managers. Trial stimulus display was 8 s. Trials were separated by inter-trial intervals (ITI) of 2 or 4 s; blocks were separated by inter-block intervals (IBI) of 10 s. During the intervals in which no stimulus was displayed, a fixation cross was shown.

For each trial in the fMRI experiment, the stimuli screen was divided into two sides depicting the choices (Figure 1). Each side included a photo of a manager, a pie chart with possible profit outcomes and respective probabilities for that manager’s proposed investment project, and expected profit for the proposed project (computed as the sum of the products of the profit outcomes in the pie chart and their probabilities). The mean expected value (EV) for the investments across all trials was $505,667, with a minimum of $486,000 and a maximum of $530,000. “Left button” or “right button” appeared above each manager’s photo at the top of the screen to remind participants to press the button that corresponded to their choice of investment project using any fingers of their left or right hands, respectively.

For trials with positive emotion managers, the project with the higher expected profit was always the one proposed by the unfamiliar manager he was paired with. For trials with a negative emotion manager, the project with the higher expected profit was always the one proposed by him rather than the unfamiliar manager he was paired with. Thus, choices based on emotions had lower expected profit, consistent with our goal to examine scenarios in which emotion regulation is required to reach economically beneficial decisions. Note, differences between EVs of the higher and lower investments in each trial were varied across trials identically for all conditions. Specifically, the mean EV difference for each of the fixed-wage and PB POS, NEG, and NEU conditions was $18,200 (SD = $6,758; range: $6,000–$30,250).

### fMRI Experimental Procedures

Stimuli presentation was controlled using E-Prime 1.2 (Psychology Software Tools, Inc., Sharpsburg, PA, USA). During the fMRI experiment, visual stimuli were back-projected onto a screen at the head of the scanner. Participants viewed the screen via an angled mirror mounted on the head coil. Participants first underwent a task orientation in a mock scanner that familiarized them with stimuli format, making choices with the button boxes, and other aspects of the scanning environment to ensure they wanted to proceed with the actual experiment. This step provided a broad overview of how the experiment would proceed but not details of the investment choice task.

After exiting the mock scanner, participants read paper-and-pencil materials instructing them to assume they were a division manager in a large organization tasked with evaluating potential investment projects proposed by other division managers that they could undertake together. This included general information on the investment proposals, their presentation format during the fMRI trials, and the time limit for each choice. Next, participants studied the materials depicting prior hypothetical interactions with six familiar managers (Supplementary Text 1). Participants then answered questions about their emotional reactions to the familiar managers (Supplementary Text 2) and had to correctly complete a quiz to match the photos of the managers with their descriptions before continuing with the task. These procedures afforded a means to ensure a successful manipulation of emotional reactions to familiar managers and task understanding before entering the scanner.

There were two phases in this blocked-design fMRI experiment. In the first, FW phase, before starting the functional runs, participants reviewed photos and summary information about the positive and negative emotion managers, and were informed they would receive $25 for the first series of 90 investment choices. Upon verifying that participants understood their task in this FW context, the functional runs began. There were three runs (each lasting 406 s) with the 90 choices distributed evenly across the runs. In each run, there were two POS, two NEG and two NEU blocks with five choice trials in each block. Runs never began with a NEU block to avoid priming participants to focus only on economic information. Block conditions within a run were presented in counterbalanced order with no consecutive occurrences of any block condition. Fixation crosses were used when no stimuli were presented. Each block lasted 54 s and was separated by 10 s with each trial displayed for 8 s separated by fixations lasting 2 or 4 s (Figure 1). In addition, 16 s fixations bounded the start and end of each run to facilitate baseline signal estimation. After completing these three functional runs, participants watched a distractor video that was chosen for engaging but not emotion-inducing content.

In the second, PB incentive, phase of the fMRI experiment, participants again reviewed photos and summary information about the positive and negative emotion managers. They were then told they would receive a PB incentive for the second series of 90 investment choices. Specifically, for a given investment choice, they could receive a bonus of 10% of profit above a threshold, to be based on four choices randomly selected out of the 90. Upon verifying that participants understood their task goal in this PB incentive context, the functional runs began. The functional runs were identical to the first FW phase except that block conditions were reordered. The order of the FW and PB incentive phases of the experiment was not varied, since completing the PB incentive phase first could have primed participants to focus on economic rather than emotional factors in their choices during a subsequent FW phase, making it difficult to identify any emotion regulation effects during the PB financial incentive phase.

### Brain Imaging Protocol

Brain images were acquired using a 3.0T Allegra system (Siemens, Erlangen, Germany) with a single-channel head coil. Echo-planar imaging (EPI) was used for each functional run, that included 203 volumes with 32 axial slices parallel to the anterior and posterior commissural plane; in-plane field of view (FOV) of 220 mm, 64 × 64 matrix; 4 mm slice thickness with 0.4 mm gap; echo time (TE) 25 ms; repetition time (TR) 2000 ms; flip-angle 90°. Co-planar high-resolution T2 anatomical scans were also acquired for functional to structural co-registration purposes; 32 slices; FOV 220 mm; 256 × 256 matrix; slice thickness 4 mm with 0.4 mm gap. For normalization of brain images to standard space, 3D magnetization-prepared rapid gradient-echo (MPRAGE) T1 structural image; 192 sagittal slices; FOV 240 mm; 192 × 192 matrix; slice thickness 1.2 mm.

### Behavioral Analysis

Given the goal of the study and the design of the POS and NEG stimuli, investment choices that were based on emotional reactions always had a lower expected profit. Thus, emotion regulation was necessary for financial incentive trials in which participants successfully chose the higher expected profit. As such, investment choices were coded 1 (0) if the project with the higher expected profit was (was not) chosen. Binary investment choices across all trials and of all participants were then used as the dependent variable in a repeated-measures logistic regression with Pay type (fixed-wage, PB) and Emotion (POS, NEG, NEU) and Pay type × Emotion as dependent variables. The resulting coefficients from this logistic regression model were then assessed for statistical evaluation of the effect of pay type and emotion on the percentage of investment choices in projects with the higher expected profit.

### Brain Imaging Analysis

Brain images were preprocessed and analyzed using SPM8 (Wellcome Trust Center for Neuroimaging, University College London). Functional images were corrected for motion and slice-time acquisition, co-registered to the MPRAGE structural image, normalized to the Montreal Neurological Institute (MNI) template brain, and smoothed with a Gaussian kernel of 8 × 8 × 8 mm.

For the whole brain analysis, a first-level general linear model was first applied to the functional data of each participant to obtain parameter estimates of each individuals’ BOLD responses in each voxel to the six investment choice block conditions: FW/positive emotion (POS_FW_); FW/negative emotion (NEG_FW_); FW/neutral emotion (NEU_FW_); PB incentive/positive emotion (POS_PB_); PB incentive/negative emotion (NEG_PB_); and PB incentive/neutral emotion (NEU_PB_). This model was based on the onsets of the different block conditions with durations of 54 s convolved with the hemodynamic response function (HRF) and also included motion correction estimates as covariates. Contrast images that estimated whole-brain voxel-wise responses for each of these six conditions relative to the implicit fixation baseline were then generated for each individual.

Individual first-level contrast images were then fed into a second-level analysis to evaluate whole-brain group responses to the different emotion conditions under FW and PB incentive contexts. We focused on two main types of whole-brain group-level responses that were relevant to our hypothesis. First, we identified voxels in which neural response differences between positive and negative emotion managers significantly differed across FW to PB incentive contexts [(POS_FW_ − NEG_FW_) – (POS_PB_ − NEG_PB_)]. Second, we identified voxels in which neural responses to emotion managers relative to neutral managers significantly differed across pay contexts separately for negative [(NEG_FW_ − NEU_FW_) – (NEG_PB_ − NEU_PB_)] and positive [(POS_FW_ − NEU_FW_) – (POS_PB_ − NEU_PB_)] cases. We expected weak and diffuse signals in this ecologically rich fMRI experiment and thus, for whole-brain contrasts, voxel primary significance threshold was first set at *p* < 0.005 (uncorrected) to improve sensitivity (Woo et al., 2014). To adjust for a whole-brain FDR of *p* < 0.05 we also applied a cluster size of at least 72 voxels based on Monte Carlo simulation with 10,000 iterations (Forman et al., 1995; Slotnick et al., 2003; Slotnick, 2011, 2017). Next, within the voxels surviving the above whole-brain criteria, we further refined our main analysis to consider only voxels that also survived a more stringent primary threshold of *p* < 0.001 and used a cluster size of at least 10 voxels based on previous studies (Lieberman and Cunningham, 2009; see Benjamini and Heller, 2007; for similar two-step approaches). Cluster-level statistics of identified brain areas are reported for both criteria.

While the whole-brain contrasts above yield brain areas that show significant differences in the modulatory effect of financial incentives on neural responses to positive and negative emotions, they cannot dissociate the following scenarios: a) enhanced neural activity for negative stimuli from fixed-wage to PB incentives relative to stable or reduced activity for positive stimuli [((NEG_PB_ − NEU_PB_) – (NEG_FW_ − NEU_FW_)) > 0, ((POS_PB_ − NEU_PB_) – (POS_FW_ − NEU_FW_)) < 0 or ≈ 0], b) reduced activity to positive stimuli from fixed-wage to PB incentives relative to stable or reduced activity for negative stimuli [((POS_FW_ − NEU_FW_) – (POS_PB_ − NEU_PB_)) > 0, ((NEG_FW_ − NEU_FW_) – (NEG_PB_ − NEU_PB_)) < 0 or ≈ 0], c) no difference in negative and positive responses for PB relative to fixed-wage responses [((POS_PB_ − NEU_PB_) – (NEG_PB_ − NEU_PB_)) ≈ 0, ((POS_FW_ − NEU_FW_) – (NEG_FW_ − NEU_FW_)) ≠ 0], and d) no difference in negative and positive responses for fixed-wage relative to PB responses [((POS_FW_ − NEU_FW_) – (NEG_FW_ − NEU_FW_)) ≈ 0, ((POS_PB_ − NEU_PB_) – (NEG_PB_ − NEU_PB_)) ≠ 0]. In particular, for the latter whole-brain contrasts where positive and negative relative to neutral manager responses are evaluated separately, it is still possible that the main effects of pay type across positive and negative emotion conditions rather than the interaction between pay type and emotion drives differences in neural responses. As such, we further evaluated conditional differences in neural responses in regions-of-interest (ROI) identified from the above whole-brain contrasts that were not afforded by the above whole-brain contrasts alone. To this end, functional ROIs were defined based on the clusters of contiguously significant voxels identified in the whole-brain contrasts and response estimates extracted from these ROIs. We then performed two-way repeated-measures ANOVAs on extracted response estimates in each ROI using Emotion (positive, negative; relative to neutral), Pay type (FW, PB incentive), and their interaction as independent variables and examined Tukey’s *post hoc* pair-wise effects. We emphasize that the ANOVAs performed on functional ROI responses were to interrogate which pair-wise effects were driving observed interactions in whole-brain contrasts and not to justify the significance of Emotion × Pay type interaction effects in the ROIs (since these are obvious from the whole-brain contrast already). For separate whole-brain contrasts of positive and negative condition responses, ROIs in which neural responses were only driven by main effects of pay type rather than the interaction between pay type and emotion were excluded from further consideration. Responses of remaining ROIs were also evaluated using Tukey’s *post hoc* pair-wise comparisons of means.

Finally, we examined how emotional neural response differences in these ROI showing significant Emotion × Pay type interaction effects were related to emotional behavioral differences in percentage of economically desirable responses. Specifically, we correlated the neural response differences between positive and negative emotions with the same emotion-related response differences in percentage choice behavior within the respective fixed-wage and PB contexts. Bonferroni adjustment was applied to the significance criterion to account for multiple comparisons across these correlations.

## Results

### Behavioral Results

Behavioral performances have previously been reported in Farrell et al. (2014) and are described briefly here. For the NEU stimuli, the proportion of higher-profit investment choices was 96.3% (SD = 8.2%) in the FW context and 95.1% (SD = 13.0%) in the PB incentive context. By contrast, the proportion of higher-profit choices for the POS stimuli was 69.3% (SD = 23.3%) in the FW context and 83.2% (SD = 19.7%) in the PB incentive context, and 63.0% (SD = 25.9%) and 82.2% (SD = 21.2%), respectively, for the NEG stimuli (Table 1). We note that these results were based on percentages pooled across all participants. Analysis using averages across participant mean choice proportions yielded similar percentages (Supplementary Text 3).

**Table 1:** Investment choice behavioral results.

Pay type	Proportion of higher-profit investment choices when unfamiliar managers paired with:		
	Neutral emotion managers	Positive emotion managers	Negative emotion managers
Fixed wage	96.3% [764 of 793]	69.3% [545 of 786]	63.0% [499 of 792]
Performance-based incentive	95.1% [759 of 798]	83.2% [660 of 793]	82.2% [656 of 798]
Percent change from fixed wage to performance-based incentive	−1.2%	20.1%	30.5%

A repeated measures logistic model with trial-wise binary investment choices as the dependent variable showed a significant Emotion (POS, NEG, NEU) × Pay type (FW, PB incentive) interaction ($\chi_{\left(1\right)}^{2}$ = 5.90* p* = 0.01), a significant main effect of Emotion ($\chi_{\left(1\right)}^{2}$ = 34.00, *p* < 0.01), but no main effect of Pay type ($\chi_{\left(1\right)}^{2}$ = 1.17, *p* = 0.28). Simple effects tests are in Table 2. Across all investment choices, across both types of pay, participants were significantly more likely to choose projects with higher expected profit with NEU stimuli than with POS and NEG stimuli (both *p* < 0.01). Pay type did not influence choices for NEU stimuli (*p* = 0.56), but did impact choices for POS and NEG stimuli (both *p* < 0.01). Moreover, there was a marginal difference in economically desirable investment choices between POS and NEG stimuli under FW ($\chi_{\left(1\right)}^{2}$ = 3.03, *p* = 0.08, two-tailed) but no difference under PB ($\chi_{\left(1\right)}^{2}$ = 0.13, *p* = 0.72, two-tailed) contexts.

**Table 2:** Simple effects tests for investment choices.

	When unfamiliar managers paired with:								
	Any emotion managers			Positive or neutral emotion managers			Negative or neutral emotion managers		
Source	Wald *χ*^2^	*df*	*p*	Wald *χ*^2^	*df*	*p*	Wald *χ*^2^	*df*	*p*
Emotion absent vs. present with:
Fixed wage	56.52	1	<0.01	49.24	1	<0.01	44.63	1	<0.01
Performance-based incentive	16.98	1	<0.01	14.04	1	<0.01	13.87	1	<0.01
Fixed wage vs. performance-based incentive with:
Emotion absent	0.35	1	0.56	0.35	1	0.56	0.35	1	0.56
Emotion present	18.92	1	<0.01	18.74	1	<0.01	14.99	1	<0.01

We also considered whether EV differences between higher and lower investments in each offer (see “Materials and Methods” section) might interact with the effect of Pay Type in modulating choice behavior associated with emotional influences. We applied a logistic regression with correct choice as the dependent variable and Pay Type, Emotion (POS, NEG, NEU), EV Difference, and their interactions as independent variables. This analysis yielded significant main effects of Emotion ($\chi_{\left(2\right)}^{2}$ = 45.0, *p* < 0.001) and EV Difference ($\chi_{\left(1\right)}^{2}$ = 19.3, *p* < 0.001), and interactions between Pay Type and Emotion ($\chi_{\left(2\right)}^{2}$ = 7.93, *p* = 0.019), and EV Difference and Emotion ($\chi_{\left(2\right)}^{2}$ = 12.3, *p* = 0.002). As would be expected, the main effect of EV Difference reflects an increase in correct choices as the difference in EV increases. The EV Difference × Emotion interaction reflects that the increase in optimal choice in response to EV Difference is greater for POS and NEG stimuli than for NEU stimuli. Additional analysis excluding neutral stimuli indicated that this increase was also greater for POS stimuli than for NEG stimuli ($\chi_{\left(1\right)}^{2}$ = 4.46, *p* = 0.035). Critically, however, EV Difference did not significantly modulate Pay Type effects on correct choices (EV Difference × Pay Type: $\chi_{\left(1\right)}^{2}$ = 0.031, *p* = 0.861; EV Difference × Pay Type × Emotion: $\chi_{\left(2\right)}^{2}$ = 2.54, *p* = 0.281.

Overall, PB incentives increased the chance that participants made economically desirable choices when emotion conflicted with economics. While this is possibly due in part to motivation to earn higher pay, it also suggests that the form of pay itself may serve as a contextual cue to regulate emotion without the need to provide explicit instructions to do so. Moreover, the effect of pay type on choice behavior differs for positive and negative reactions, which was stable across differences between the EVs of investment choices.

### Differential Neural Responses to Positive and Negative Emotion Managers across Pay Contexts

**Figure 2:**
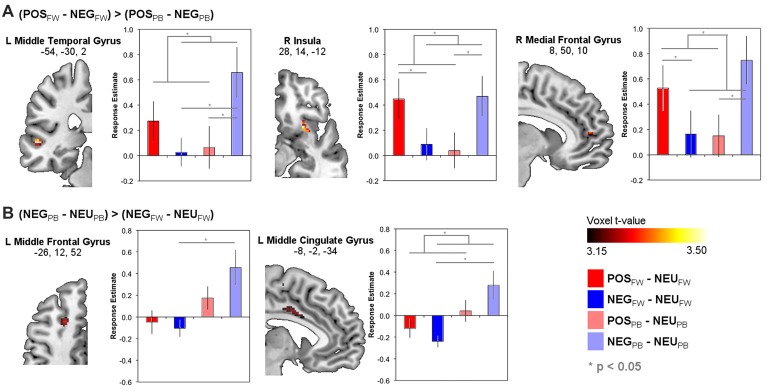
Brain functional response estimates to positive and negative emotional relative to neutral stimuli during FW and PB incentives in regions-of-interest (ROI) identified from whole-brain contrasts that showed significant modulations of responses to emotions across pay type contexts (see Table 3). **(A)** Brain areas showing direct differences between neural responses to positive and negative emotional stimuli that differed across pay type. **(B)** Brain areas showing changes in neural responses to negative emotional relative to neutral stimuli across pay type. Statistical overlays on brain slices are thresholded at *p* < 0.001 (uncorrected) with a cluster size > 10 voxels (see “Materials and Methods” section regarding significance criteria details).

Supplementary Table S1 lists brain areas with significant POS and NEG responses relative to NEU for fixed-wage and PB contexts separately. Importantly, whole-brain analysis of regions in which the functional response differences between POS and NEG stimuli differed between FW and PB incentive contexts are reported in Table 3 and depicted in Figure 2. As seen in Figure 2A, this analysis yielded the left middle temporal, right insula, and medial frontal areas, in which response differences between POS relative to NEG stimuli were greater during FW than PB incentive contexts. Repeated measures ANOVA of neural responses in functional ROIs from the above whole-brain contrast validated that all these regions evinced the expected significant Emotion × Pay type interactions (see Supplementary Text 4) and, additionally, showed no significant main effects of Emotion or Pay type. *Post hoc* Tukey pair-wise results are depicted in Figure 2 and highlighted here. Critically, in all these ROIs, we observed a double-dissociation in that *decrease* in POS stimuli responses from FW to PB incentive contexts was significantly distinct from the *increase* in responses to NEG stimuli ((POS_FW_ − POS_PB_) – (NEG_FW_ − NEG_PB_)); left middle temporal: *t*_(23)_ = 2.61, *p* = 0.016; right insula: *t*_(23)_ = 3.06, *p* = 0.005; right medial frontal: *t*_(23)_ = 2.55, *p* = 0.018). In the FW condition, responses to POS stimuli were higher than to NEG stimuli in all ROIs (POS_FW_ − NEG_FW_; right insula: *t*_(23)_ = 2.34, *p* = 0.028; right medial frontal: *t*_(23)_ = 2.53, *p* = 0.019), although this did not reach significance in the left middle temporal region (*t*_(23)_ = 1.81, *p* = 0.084). In the PB condition, responses to NEG stimuli were significantly higher than to POS stimuli in all ROIs (POS_PB_ − NEG_PB_; left middle temporal: *t*_(23)_ = −2.63, *p* = 0.015; right insula: *t*_(23)_ = −2.37, *p* = 0.028; right medial frontal: *t*_(23)_ = −2.10, *p* = 0.047). These findings suggest that brain regions identified here processed the valence of the stimuli relevant to the pay type contexts whether the valence stemmed from emotional or economic benefit.

**Table 3:** MNI peak coordinates of brain areas that showed significant differences in functional responses to emotional stimuli across pay type conditions that additionally showed significant Emotion (POS-NEU, NEG-NEU) × Pay type (FW, PB) interactions in region-of-interest (ROI) analysis performed on that cluster (see “Materials and Methods” section).

Contrast	Brain region	BA	*x*	*y*	*z*	*T*	*p* < 0.005, *k* > 72		*p* < 0.001, *k* > 10	
							No. of voxels	Cluster p(unc.)	No. of voxels	Cluster p(unc.)
(POS_FW_ − NEG_FW_) > (POS_PB_ − NEG_PB_)	L Middle Temporal Gy.	22	−54	−30	2	3.56	200	0.086	38	0.302
	R Insula	48	28	14	−12	3.45	140	0.144	25	0.404
	R Medial Frontal Gy.	32	8	50	10	3.33	239	0.063	16	0.510
(NEG_PB_ − NEU_PB_) > (NEG_FW_ − NEU_FW_)	L Middle Frontal Gy.	8	−26	12	52	3.20	448	0.015	57	0.208
	L Middle Cingulate Gy.	24	−8	−2	34	3.70	611	0.006	113	0.084

*Gy.: Gyrus. POS, NEG, or NEU = positive, negative, or neutral emotion manager, respectively; FW, PB = fixed wage or performance-based incentive, respectively. Cluster-level voxel counts and p-values are indicated for both whole-brain p_(FDR)_ < 0.05 (voxel p < 0.005, k > 72) and for voxel p(unc.) < 0.001, k > 10 criteria (see “Materials and Methods” section)*.

Brain regions in which functional responses to POS or NEG stimuli relative to NEU stimuli separately differed across pay type are in Figure 2B, Table 3 and Supplementary Table S2. For NEG stimuli, this analysis yielded significant pay type effects on neural responses in the left middle frontal, middle cingulate, supplementary motor, hippocampus, and visual areas, as well as bilateral caudate. Of these, evaluation of neural responses in these identified functional ROIs using ANOVA found significant Emotion × Pay type interactions only in the left middle frontal (*F*_(1,88)_ = 13.0, *p* = 0.001) and middle cingulate (*F*_(1,88)_ = 5.04, *p* = 0.27) regions (Figure 2B), albeit there were significant main effects of Pay type (left middle frontal: *F*_(1,88)_ = 20.9, *p* < 0.001; left middle cingulate: *F*_(1,88)_ = 12.5, *p* = 0.001) and no main effects of Emotion. Specifically, while neural responses in these two ROIs generally increased from FW to PB incentive contexts, this increase was larger in magnitude for NEG than POS managers (left middle frontal: POS_FW_ − POS_PB_, *t*_(32)_ = −1.83, n.s.; NEG_FW_ − NEG_PB_, *t*_(32)_ = −3.07, *p* = 0.005; left middle cingulate: POS_FW_ − POS_PB_, *t*_(32)_ = −1.18, n.s.; NEG_FW_ − NEG_PB_, *t*_(32)_ = −3.75, *p* = 0.001; further (POS_FW_ − POS_PB_) – (NEG_FW_ − NEG_PB_), *t*_(32)_ = 2.11, *p* = 0.04). For POS stimuli, pay type effects were evident in the right parahippocampal and left fusiform areas with ROI analyses of these areas yielding no significant Emotion × Pay type interactions. Thus, the left middle frontal and middle cingulate regions engaged greater neural processing effort in PB than fixed-wage contexts for NEG but not POS stimuli, suggesting their involvement in regulating negative emotions.

### Emotion-Related Neural Responses in ROIs Correlated with Investment Choice Task Performance

**Figure 3:**
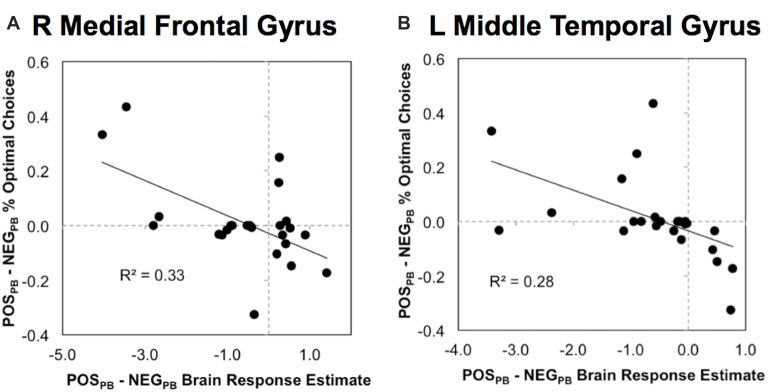
Scatterplots of significant correlations between functional responses and investment behavioral choices in **(A)** R medial frontal gyrus and **(B)** L middle temporal gyrus in which pay type modulated emotional responses (see Figure 2). Y-axes are proportions of economically-desirable choices in PB pay context. POS_PB_ − NEG_PB_ % Optimal Choices indicates proportion when positive emotion is present less proportion when negative emotion is present. Brain response estimates on x-axes indicate magnitude of brain response differences between emotional during PB pay context (POS_PB_ − NEG_PB_).

Finally, we examined how differential functional responses to POS and NEG stimuli in the ROIs above showing significant Emotion × Pay type interactions correlated with emotional-related effects on investment choices within fixed-wage and PB contexts. Bonferroni adjustment on the significance criterion was based on the 10 correlations across five ROIs and two neurobehavioral indices (POS_FW_ − NEG_FW_ and POS_PB_ − NEG_PB_). Significant brain-behavior correlations were found in the right medial frontal and left middle temporal regions for emotional response differences during the incentive-based context (Figure 3). In the right medial frontal ROI, higher brain responses to POS relative to NEG stimuli correlated with poorer performances for POS relative to NEG stimuli during the PB context (*r* = −0.574, *n* = 24, *p* = 0.003, two-tailed; Figure 3A). Similar correlations were seen in the left middle temporal ROI (*r* = −0.527, *n* = 24, *p* = 0.008, two-tailed; Figure 3B). These findings suggest that higher sensitivity to POS than NEG stimuli right medial frontal and left middle temporal regions during the PB context corresponded with less economically desirable investment choices.

## Discussion

Under a flat wage that provides minimal incentive to regulate emotions, middle temporal, insula, and medial prefrontal regions engaged higher neural responses to positive than negative emotional decisions, despite associations with lower economically desirable outcomes in the former. Such primacy in neural affective responses reflects a mechanism for the influence of conflicting emotional information on economic decisions (Kida et al., 2001; Moreno et al., 2002; Thaler et al., 2013). Critically, with PB incentives negative emotional decisions evoked greater neural activity than positive emotional decisions in frontal and cingulate regions, with the hippocampal and caudate regions additionally showing higher activity during negative than neutral emotional decisions. These findings reflect different neural processes during value-based decisions that underlie the motivation of individuals to seek out positive emotional subjective experiences and the avoidance of negative ones rather than to make financially sound objective judgments. Importantly, this primary motivation can be circumvented when competing incentives have personal relevance to the individual, albeit with the recruitment of more cognitive effort, especially for negative emotions.

Several studies have reported similar brain areas consistent with ours involved in processing affective information particularly for facial expressions of others (Buckner et al., 2008; Suzuki et al., 2011; Phelps et al., 2014). Specifically, greater activity in the temporal and insula regions, amongst others, is observed when participants undergo tasks that involve empathy for facial emotions (Carr et al., 2003). Thus, it is possible that higher neural responses to positive managers in our task may have reflected a sense of empathy in participants, biasing them to make decisions in favor of these managers. Medial frontal and insula processing of emotional stimuli should be considered in tandem with its involvement in value-based decision-making. Neural activity in these region are modulated in response to stimuli that are perceived to be more valuable or more costly (Knutson et al., 2001, 2005; Lim et al., 2011; Bartra et al., 2013). Such co-variation of activity with stimuli value in the medial frontal region and insula might be a neural mechanism for the incorporation of stimuli information from other brain areas into an integrated subjective value that then influences decision behavior. We suggest that because there is no personal financial cost to participants under a FW, subjective value is more directly linked to the affective features of stimuli that are considered desirable to the participant.

Under PB incentives, however, participants have to consider two streams of potential subjective value—the affective aspect in contrast to the financial aspect, which now also has competing personal relevance. In this context, we found higher neural responses to negative than positive stimuli in the above affective processing regions as well as the left middle frontal and middle cingulate gyri. Also, hippocampal and caudate regions were more active during negative than neutral conditions. We note that the key difference between the fixed-wage and PB contexts is a change in the criterion for subjective value. Negative emotion managers, which are bound to the more economically desirable options, are then subjectively more valuable under PB incentives than the positive emotion managers. Indeed, neural responses in the affective processing regions were higher to negative stimuli compared to positive or neutral stimuli in the PB context.

However, such a switch in the subjective value of the same stimuli from fixed-wage to PB incentives is insufficient to explain the additional engagement of left middle frontal and middle cingulate as well as hippocampal and caudate regions specifically to negative emotional stimuli. Rather, the involvement of these regions during negative emotional conditions suggests that, apart from reversals in stimuli valuation in the affective systems, participants were engaging additional regulatory processes in order to arrive at a decision. Indeed, much work focuses on techniques that incorporate explicit instructions to regulate emotion such as expressive suppression and cognitive reappraisal (Ochsner and Gross, 2008; Koole, 2009; Gross, 2013). Examinations of the neural correlates underlying such explicit emotion regulation techniques have found increased activity in regions of the prefrontal cortex, anterior cingulate, and also parietal areas, which likely signals increased inhibitory processing (Ochsner et al., 2012). Our study extends these findings by showing that contextual features, such as PB financial incentives, might also implicitly evoke processes related to emotion regulation in these middle frontal and cingulate regions in order to meet task goals. In addition, we observed that hippocampal and caudate regions also modulated their activity to negative emotion managers. Given the role of these structures in associative learning (Schultz and Dickinson, 2000; Squire et al., 2004; Haruno and Kawato, 2006; Suzuki, 2007), we speculate that the additional activity in these regions might reflect top-down signaling of significant unpredicted events—accepting offers given by negative managers.

It is tempting to further consider pay type and emotional influences on neural processes at the trial-wise level that underlie making suboptimal or optimal choices. However, we designed our study to evaluate general effects of pay type on rejecting subpar investments with positive affect and accepting optimal investments with negative affect. As such, we had limited statistical power and reliability for fair comparisons of neural responses during rejected and accepted trials under both positive and negative affect conditions. We also did not observe significant interactions between specific trial-wise EV differences on the effect of pay type on investment choice behaviors. Thus, further studies specifically manipulating the balance of optimal and suboptimal choices participants make and the effects of stimuli EV are necessary to teasing apart such fine-level trial-wise neural computations. Nevertheless, we highlight that our work is distinct from two other streams of neuroscience research that link financial rewards and emotion regulation. One stream examines whether and how explicit emotion regulation instructions can impact the emotions elicited by the anticipation or receipt of monetary rewards or losses (e.g., Knutson et al., 2001; Staudinger et al., 2011; Kirk et al., 2015). The other stream uses tasks such as dictator or ultimatum games in which individuals must determine how to share monetary rewards to examine brain regions associated with explicit emotion regulation strategies like reappraisal (Grecucci et al., 2013a,b). In contrast to these, we examine the neural mechanisms of how monetary rewards themselves regulate positive and negative emotions using a context-rich and ecologically valid fMRI experimental task.

One additional consideration regarding our task implementation is whether the order of presenting fixed-wage followed by PB incentives might matter for behavioral and brain responses. For instance, our findings of choice changes during PB contexts might be due more to adaptation from simply having made similar decisions during the earlier fixed-wage context, than the PB manipulation itself. We point interested readers to details of our consideration of this issue in our prior publication, which found minimal evidence for such adaption effects (Farrell et al., 2014). Briefly, we found that participants had longer response times for the second PB compared to the first fixed-wage contexts, longer response times for emotional compared to neutral managers, with no interactive effect between contract type and emotion managers on response times. Moreover, in this present study, we also found a double dissociative effect on neural responses between positive and negative emotional managers. Specifically, whereas responses in middle temporal, insula, and medial frontal areas decreased from POS_FW_ to POS_PB_, responses increased from NEG_FW_ to NEG_PB_. Further, Supplementary Figure S1 details additional examinations of brain response difference between the first and second halves of the experiment. Note that these analyses showed higher neural responses during PB (second half) compared to fixed-wage (first half) for emotional relative to neutral conditions. Taken together, we suggest that it is difficult to account for our behavioral and brain findings based on simple adaptation-related reduction in neural processing due to repeated visual stimuli exposure effects alone. Also, while it is interesting to examine differences if PB incentives were applied first, we reasoned that it is likely difficult for participants to “un-regulate” emotions for FW performance after the PB context. Thus, we suggest that our implementation is more informative for assessing brain activity under the FW contract as a baseline for unregulated emotion, and evaluating if indeed PB incentives serve as an emotion regulation device.

There is extensive attention in psychology and neuroscience research on how different emotion regulation techniques moderate the impact of emotions on choice (Phelps et al., 2014; Lerner et al., 2015). In daily life, however, individuals are rarely explicitly instructed to regulate their emotions (Silvers et al., 2015), and even when they are, they may not use the suggested techniques (Suri et al., 2015). As an alternative to explicit emotion regulation, contextual elements of decision environments can be adjusted to implicitly influence individuals toward choices that require regulating emotions (Thaler et al., 2013; Lerner et al., 2015). For instance, financial incentives linked to monetary outcomes might serve as a contextual influence to regulate emotional reactions by priming participants to financial considerations in decisions (Lerner et al., 2015). Indeed, our findings show that provision of personally relevant benefits or costs in decision contexts is sufficient to evoke additional neural processes that bypass the primary motivation of human individuals to seek positive emotional experiences and avoid negative ones.

The human brain appears to be wired to use the least amount of neural resources possible to achieve circumstances that are as subjectively favorable as possible—a lazy and selfish brain. Thus, in some ways, it is not surprising that organizational research and practice have long recognized that emotions negatively impact job attitudes, behaviors, and choice in the workplace (Brief and Weiss, 2002; Barsade and Gibson, 2007), and that the instantiation of personally relevant PB financial incentives can help to regulate emotional influences on decisions. In this light, our study identifies where and how affective and regulatory processes operate in the brain so that financial incentives differentially modulate decisions associated with positive and negative emotion. Understanding such brain mechanisms for accommodating specific competing environmental influences in individual choices may be instrumental in identifying factors that motivate decisions which result not only in subjective gains for the individual, but organizational benefits as well.

## Author Contributions

AMF, BJW and JOSG: conceptualization, methodology and writing; AMF and BJW: investigation; BJW and JOSG: formal analysis; AMF: funding acquisition and resources.

## Conflict of Interest Statement

The authors declare that the research was conducted in the absence of any commercial or financial relationships that could be construed as a potential conflict of interest. The reviewer JL and handling Editor declared their shared affiliation, and the handling Editor states that the process nevertheless met the standards of a fair and objective review.
