# Supplementary material for: Financial Incentives Differentially Regulate Neural Processing of Positive and Negative Emotions during Value-Based Decision-Making
Source: Front Hum Neurosci. 2018 Feb 13;12:58. doi: 10.3389/fnhum.2018.00058 (PMC5816803; doi:10.3389/fnhum.2018.00058)

Financial Incentives Differentially Regulate Neural Processing of Positive and Negative Emotions During Decision-Making

Supplementary Materials

Table of Contents

	Description
Supplementary Text 1	Sample inducement of emotional reaction
Supplementary Text 2	Pre-and post-scanner questions about emotional reactions and analysis of responses
Supplementary Text 3	Comparison between pooled data and means per participant.
Supplementary Table 1	Peak activity table of whole-brain contrasts of neural responses to positive and negative relative to neutral manager stimuli across fixed-wage and performance-based conditions. Cluster-level voxel counts and p-values are indicated for voxel primary threshold set at p(unc.) < 0.001, k > 10, although all areas survived the whole-brain p(FDR) < 0.05 criteria (see Methods).
Supplementary Text 4	Statistical values for repeated measures ANOVA on functional ROIs.
Supplementary Table 2	Additional results of whole-brain analysis evaluating changes in neural responses across fixed-wage to performance-based conditions separately for positive and negative emotion (relative to neutral) conditions, and repeated measures ANOVA of the functional ROI responses. Cluster-level voxel counts and p-values are indicated for voxel primary threshold set at p(unc.) < 0.001, k > 10, although all areas survived the whole-brain p(FDR) < 0.05 criteria (see Methods).
Supplementary Fig. 1	Axial slices depict whole-brain contrast (POSFW + NEGFW - 2*NEUFW) - (POSPB + NEGPB - 2*NEUPB) in red that shows neural response differences between the 1st and 2nd halves of the experiment (which were also the FW and PB conditions, respectively) relative to the respective baselines. Only higher responses to PB than FW were observed in left fusiform, parahippocampal, right caudate, temporal, and middle cingulate areas. Whole-brain contrast (POSFW + NEGFW + NEUFW) - (POSPB + NEGPB + NEUPB) in blue shows overall brain activity level differences between 1st and 2nd halves of the experiment. Bilateral frontal, parietal, temporal and visual areas, not overlapping with emotion-related response areas in a) or in our main analyses, showed higher responses during FW than PB with no reverse effects. Statistical overlay threshold was set at p < 0.001, cluster-size > 10.

Supplementary Text 1. Sample inducement of emotional reaction, from Farrell *et al.* (2014). We induced positive or negative emotional reactions to hypothetical managers. Below is negative emotion inducement ("Barry") and a positive emotion inducement ("John"). Photos are removed due to copyright issues.

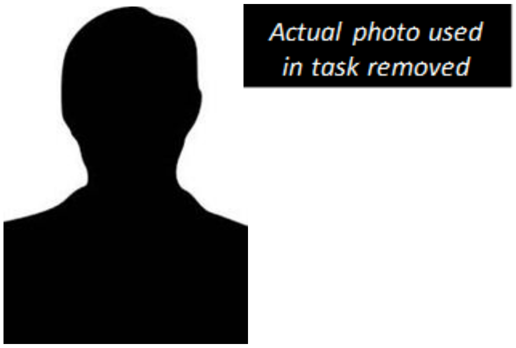

Barry

You have worked with Barry on several projects. Each time, working with him was extremely stressful and unpleasant, for a number of reasons. First, Barry is a procrastinator. He leaves important tasks that he agrees to complete until the last possible moment, leaving others that are waiting for his work without a "cushion" if things on their end should go wrong. This makes everyone working on a project with him (including you) extremely anxious. More than once, Barry's procrastination has caused others in the organization to have to double their efforts and work long hours to stay on target.

Second, Universal's culture is very cooperative, with everyone assigned to a project pitching in to get the job done. However, no one considers Barry a "team player", and in fact he can be downright uncooperative and detrimental to a project. For example, your team and Barry's recently partnered on a project to bring a new product to market. When you encountered a major flaw in the proposed manufacturing process that had the potential to significantly delay the product's introduction, you and your team dug in and worked long hours to find a fix, and your understanding was that Barry's team was doing the same. However, when you called Barry to set up a meeting to discuss a possible solution your team had identified, his assistant informed you that he had left the previous day on a golfing vacation. When you finally reached Barry, he said he had told his team the problem wasn't that big of a deal, it was your problem anyway, and it could wait until he returned from vacation the following week; he rushed off the phone to "get back to the golf course". In the end, you and your team found a solution to the problem, but Barry's lack of concern, unwillingness to pitch in, and lack of appreciation for the efforts of others is typical of his approach to "teamwork".

Third, Universal's division managers make a real effort to get to know their colleagues both professionally and personally. This includes remembering each others' interests so you can engage in meaningful and enjoyable conversations about non-work topics, knowing the names of close family members, sending small gifts to celebrate successes in the firm, and providing support if colleagues are having a tough time at work or at home. Since you and Barry have worked together several times, you have made a special effort to be just this kind of colleague. Barry, however, takes no interest in his colleagues, and in conversations about both work and home he is aloof and disinterested. In fact, he has trouble remembering your name, much less those of your team members or family members.

While you obviously do not like these qualities, you do not believe they impact the overall amount of work you or your team do on projects you do with Barry. However, in short, Barry is a procrastinator and an uncooperative colleague, and is rude in his interactions with others.

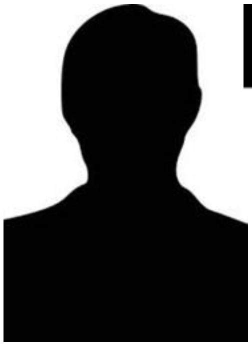

Actual photo used in task removed

John

You have been best friends with John since college. You have always enjoyed spending time with him because of his great sense of humor and personable, easy-going nature. Further, you share many of the same values and have similar likes and dislikes. In college, you and John were housemates for three years and had the same major. You associated with the same group of friends who even traveled together on vacation breaks. Just after graduation, you and John and some of these friends went on an eight-week backpacking trip throughout Europe which you still fondly remember today. After college, even though you lived in different parts of the country, you and John continued to socialize and travel together regularly with a group of mutual friends. You were in each other's weddings, shared in the joys when children arrived, and shared stories of your highs and lows in your careers and social lives. In fact, John introduced you to the person you would later marry.

Your families also get along very well, and in fact happily vacation together for two weeks every summer in Maine. Several years ago, you were pleased when you heard John was looking for a job, as you knew Universal would be a perfect fit for him. When John told you he had taken a position as a division manager at Universal, you were so thrilled that you immediately told your spouse and kids, who were equally as happy to have John and his wife moving to your town. He quickly became an active, respected member of your community, involved in volunteer work and coaching his, and your, children's soccer teams.

Through the years, your friendship has grown stronger, and you consider John to be one of your best friends. He has consistently been a positive and supportive individual, both personally and professionally. No matter how busy life has gotten for either of you, John has always been there, and you have no doubt your friendship with John will continue to grow.

Not surprisingly to you, within Universal John has an excellent reputation. Others who have worked with him remark on his trustworthiness and honesty, and the fact that he treats everyone, from the night watchman to the CEO, with the same respect. He is known to be an effective division head who helps his staff grow into future leaders of the firm. He is extremely well-liked by all who work with him, and all who know him.

In short, John is your best friend, and is trustworthy and well-liked within the firm.

Supplementary Text 2. Pre-and post-scanner questions about emotional reactions and analysis of responses.

For each of the positive and negative emotion managers, participants answered these questions before entering and again after leaving the scanner.

	Strongly disagree					*Neither agree nor disagree*					*Strongly agree*
I was happy with {insert name}.	0	1	2	3	4	5	6	7	8	9	10
I was angry with {insert name}.	0	1	2	3	4	5	6	7	8	9	10
I was afraid of {insert name}.	0	1	2	3	4	5	6	7	8	9	10
I liked {insert name}.	0	1	2	3	4	5	6	7	8	9	10
I was frustrated with {insert name}.	0	1	2	3	4	5	6	7	8	9	10
I was elated with {insert name}.	0	1	2	3	4	5	6	7	8	9	10
	Strongly disagree					*Neither agree nor disagree*					*Strongly agree*

Based on the following tests, the inducements of emotional reactions resulted in the desired responses from participants and were sustained for the duration of the task.

- We compared participants' mean responses for three measures of positive emotion (happy, liked, elated) and two measures of negative emotion (anger, frustration) to the 5.0 scale midpoint. Both before and after completion of the investment choice task:
  - For the positive emotion managers, the mean response to the positive (negative) emotion questions of 8.65 (0.32) was significantly above (below) the midpoint (all $p < 0.05$, one-tailed).
  - For the negative emotion managers, the mean response to the positive (negative) emotion questions of 2.06 (6.39) was significantly below (above) the midpoint (all $p < 0.05$, one-tailed).
  - For all emotion managers, consistent with expectations that we would not induce fear, the mean responses to the fear question were significantly lower than the midpoint (all $p < 0.01$, one-tailed).
  - Note, we used one-tail tests here since we expected a specific directional difference. For example, for questions about positive emotional reactions to the hypothetical managers, we expect higher responses (denoting stronger agreement with the statement) for the "positive emotion" managers than for the "negative emotion" managers.

- We compared mean responses before and after completion of the investment choice task.
  - For the positive emotion managers, the mean response to the positive emotion questions was higher before (8.96) than after (8.36) the task, but still higher than the scale midpoint ($p = 0.01$, two-tailed). Thus, reactions dissipated but were still sufficiently high.

- For the negative emotion managers, the mean response to the negative emotion questions was not different before (6.57) and after (6.20) the task ($p = 0.24$, two-tailed).

Note, we used the above questions to test whether we successfully induced emotional reactions to the two "positive emotion" and two "negative emotion" managers. As such, we did not ask these same questions of the two "neutral emotion" managers. Nevertheless, the responses suggest we successfully induced significantly different reactions to the "positive emotion" and "negative emotion" managers, and we provided one-sentence backstories for the "neutral emotion" managers ("Here are managers with whom your interactions have been fairly ordinary") compared to lengthy backstories for the "positive" and "negative emotion" managers (illustrated in Supplementary Text 1).

Supplementary Text 3

We note that the percentage profitable choices reported in Table 1 are based on data pooled over all participants. It is possible that averaging over the mean percentage for each participant might yield different results. However, we note that these two methods give equal means as long as all participants answered all questions. We had a very small number of non-responses (around 2 percent). As a result, there was virtually no difference between the mean percentages using the two methods, as shown in the tables below. We opted to present the pooled results, as this more accurately reflects the number of observations that are used in the statistical analysis (while modeling the non-independence of multiple responses from the same subject vis-à-vis repeated measures logistic regression).

Table 1a: Pooling all responses

	Neutral	Positive	Negative
Fixed Wage	96.3%	69.3%	63.0%
Performance-based	95.1%	83.2%	82.2%

Table 1b: First calculate mean per participant, then take the mean of these means

	Neutral	Positive	Negative
Fixed Wage	96.3%	69.1%	62.9%
Performance-based	95.1%	83.3%	82.2%

Supplementary Table 1. Peak activity table of whole-brain contrasts of neural responses to positive and negative relative to neutral manager stimuli across fixed-wage and performance-based conditions. Cluster-level voxel counts and p-values are indicated for voxel primary threshold set at p(unc.) < 0.001, k > 10, although all areas survived the whole-brain p(FDR) < 0.05 criteria (see Methods).

Contrast	Brain Region	BA	x	y	z	T	No. of voxels	Cluster p(unc.)
POS_BP - NEU_BP	R Angular Gyrus	39	50	-62	38	6.49	903	0.000
	L Angular Gyrus	39	-52	-68	34	6.29	1074	0.000
	R Posterior Cingulate Gyrus	23	10	-48	26	5.34	1249	0.000
	R Superior Medial Frontal Gyrus	32	2	36	30	4.96	1472	0.000
	R Temporal Pole	20	52	4	-34	4.66	156	0.046
	L Superior Frontal Gyrus	9	-14	36	52	4.14	107	0.092
	R Middle Cingulate Gyrus	23	2	-22	36	4.11	96	0.108
	L Anterior Insula	48	-28	14	-10	3.98	57	0.208
	R Anterior Insula	48	30	14	-10	3.96	43	0.272
	L Temporal Pole	20	-44	6	-38	3.73	40	0.289
	R Superior Frontal Gyrus	9	20	32	50	3.55	29	0.368
	R Middle Frontal Gyrus	46	36	20	44	3.50	18	0.483
	R Inferior Frontal Gyrus Pars Orbitalis	47	46	28	-8	3.48	23	0.424
	L Orbitofrontal Gyrus	47	-38	38	-10	3.42	13	0.556
	L Inferior Frontal Gyrus Pars Orbitalis	47	-42	20	-10	3.41	12	0.574
	L Middle Frontal Gyrus	46	-28	20	44	3.39	11	0.592
	R Entorhinal Area	36	30	4	-32	-4.80	51	0.233
	L Hippocampus	20	-36	-26	-16	-4.43	68	0.171
	L Superior Frontal Gyrus	6	-12	-6	70	-4.10	43	0.272
	R Superior Frontal Gyrus	6	12	-6	70	-3.99	65	0.180
	R Precentral Gyrus	6	38	-12	48	-3.97	135	0.061
	L Postcentral Gyrus	2	-20	-42	66	-3.86	74	0.154
	R Postcentral Gyrus	2	30	-38	62	-3.62	33	0.336
	L Superior Occipital Gyrus	18	-22	-88	14	-3.58	133	0.063
	L Fusiform Gyrus	18	-24	-76	-2	-3.50	49	0.242
	R Postcentral Gyrus	2	14	-38	66	-3.44	31	0.351

Supplementary Table 1 (cont'd)

Contrast	Brain Region	BA	x	y	z	T	No. of voxels	Cluster p(unc.)
NEG_BP - NEU_BP	R Angular Gyrus	39	52	-62	38	5.97	725	0.000
	L Angular Gyrus	39	-52	-64	26	4.40	507	0.001
	L Precuneus	7	-2	-62	38	4.22	339	0.006
	R Temporal Pole	20	50	2	-36	3.87	25	0.404
	L Middle Temporal Gyrus	21	64	-16	-18	3.84	47	0.251
	L Superior Frontal Gyrus	8	-12	30	56	3.82	33	0.336
	L Temporal Pole	20	-46	2	-38	3.76	25	0.404
	L Superior Medial Frontal Gyrus	10	-2	58	12	3.46	13	0.556
	L Superior Medial Frontal Gyrus	10	0	52	28	3.36	28	0.376
	R Posterior Cingulate Gyrus	23	8	-48	28	3.28	12	0.574
	L Superior Frontal Gyrus	6	-12	-8	70	-4.51	136	0.061
	R Precentral Gyrus	4	32	-18	52	-4.45	856	0.000
	L Superior Parietal Lobule	5	-14	-44	64	-4.38	428	0.002
	L Fusiform Gyrus	37	-38	-48	-14	-4.12	98	0.105
	L Superior Occipital Gyrus	18	-18	-86	10	-4.09	109	0.089
	L Inferior Frontal Gyrus Pars Orbitalis	11	22	26	-8	-3.85	25	0.404
	L Precentral Gyrus	6	-42	-12	50	-3.73	52	0.228
	R Superior Frontal Gyrus	6	12	-6	70	-3.72	45	0.262
	R Inferior Occipital Gyrus	37	42	-64	-2	-3.62	24	0.414
	R Superior Occipital Gyrus	19	26	-76	22	-3.48	51	0.233
	L Middle Occipital Gyrus	19	-32	-86	20	-3.40	50	0.237
POS_AP - NEU_AP	R Middle Frontal Gyrus	46	32	18	38	4.70	527	0.001
	L Precuneus	7	-4	-66	42	4.44	1050	0.000
	L Angular Gyrus	39	-54	-60	32	4.21	600	0.001
	L Middle Frontal Gyrus	44	-34	20	32	4.16	235	0.018
	L Superior Frontal Gyrus	8	-10	32	54	4.00	152	0.049
	R Angular Gyrus	22	56	-54	28	3.97	371	0.004
	R Superior Frontal Gyrus	8	10	30	56	3.79	147	0.052
	L Thalamus	-	-10	-8	6	3.64	50	0.237

Supplementary Table 1 (cont'd)

Contrast	Brain Region	BA	x	y	z	T	No. of voxels	Cluster p(unc.)
	L Superior Frontal Gyrus	9	16	44	42	3.42	16	0.510
NEG_AP - NEU_AP	R Superior Frontal Gyrus	32	12	28	48	4.93	3607	0.000
	R Middle Temporal Gyrus	21	46	-36	-2	4.38	352	0.005
	L Middle Temporal Gyrus	21	-50	-36	-6	4.37	650	0.000
	L Precuneus	7	-2	-56	40	4.13	461	0.002
	L Angular Gyrus	39	-48	-56	28	3.92	419	0.003
	L Inferior Frontal Gyrus Pars Orbitalis	47	-46	32	-12	3.74	56	0.212
	R Angular Gyrus	22	54	-52	24	3.66	65	0.180
	R Inferior Frontal Gyrus Pars Orbitalis	47	44	24	-10	3.64	29	0.368
	L Thalamus	-	-8	-4	12	3.61	82	0.135
	R Middle Frontal Gyrus	9	40	8	46	3.57	25	0.404
	L Precentral Gyrus	6	-42	2	46	3.53	34	0.329
	R Caudate	-	16	-4	20	3.51	24	0.414
	L Temporal Pole	21	-52	8	-30	3.44	22	0.435

Supplementary Text 4

Neural responses in functional ROIs from the $[(POS_{FW} – NEG_{FW}) – (POS_{PB} – NEG_{PB})]$ whole-brain contrast were submitted to a repeated measures ANOVA using Emotion (POS – NEU, NEG – NEU) and Pay type (FW, PB) as independent variables, and statistical values for the Emotion × Pay type interaction are listed as follows. Left middle temporal: $F(1, 88) = 7.66$, $p = 0.007$; right insula: $F(1, 88) = 4.67$, $p = 0.033$; right medial frontal: $F(1, 88) = 6.01$, $p = 0.016$). Because the whole-brain contrast already identified the above voxels as showing significant Emotion × Pay type interaction effects, these F and p values are for reference only. Critical effects focus on post-hoc pair-wise comparisons reported in the main text.

Supplementary Table 2. Additional results of whole-brain analysis evaluating changes in neural responses across fixed-wage to performance-based conditions separately for positive and negative emotion (relative to neutral) conditions, and repeated measures ANOVA of the functional ROI responses. Cluster-level voxel counts and p-values are indicated for voxel primary threshold set at p(unc.) < 0.001, k > 10, although all areas survived the whole-brain p(FDR) < 0.05 criteria (see Methods).

Contrast	Brain Region	BA	x	y	z	T	No. of voxels	Cluster p(unc.)	F		
									Emotion	Pay type	Emotion x Pay type
$(NEG_{PB}\text{-}NEU_{PB}) > (NEG_{FW}\text{-}NEU_{FW})$	L Supplementary Motor Area	6	-12	-6	62	4.20	111	0.086	0.34	26.7***	2.89
	L Caudate	-	-18	32	0	4.01	41	0.284	0.34	7.35**	1.70
	R Caudate	-	18	30	0	3.95	120	0.076	0.05	4.31*	2.51
	L Middle Occipital Gyrus	37	-40	-62	6	3.86	58	0.204	1.00	8.03**	1.14
	L Fusiform Gyrus	37	-40	-44	-22	3.80	68	0.171	0.11	15.6***	2.82
	L Hippocampus	20	-36	-28	-14	3.63	20	0.458	1.88	11.5**	1.63
$(POS_{PB}\text{-}NEU_{PB}) > (POS_{FW}\text{-}NEU_{FW})$	R Parahippocampal Gyrus	36	30	6	-32	4.62	59	0.201	0.157	7.30**	0.546
	L Fusiform Gyrus	20	-36	-26	-16	3.91	33	0.336	1.10	10.3**	0.913

Note: MNI peak coordinates of brain areas that showed significant differences in functional responses to negative and positive stimuli across pay type conditions but no significant Emotion (POS-NEU, NEG-NEU) x Pay type (FW, PB) interactions in ROI analyses. The lack of interaction effects in these ROIs indicate that while functional responses within negative or positive emotion conditions differed over pay type conditions, these differences were not significantly dissociated between emotion.

*p < 0.05, **p < 0.01, ***p < 0.001.

Supplementary Fig. 1. Axial slices depict whole-brain contrast (POSFW + NEGFW - 2*NEUFW) - (POSPB + NEGPB - 2*NEUPB) in red that shows neural response differences between the 1st and 2nd halves of the experiment (which were also the FW and PB conditions, respectively) relative to the respective baselines. Only higher responses to PB than FW were observed in left fusiform, parahippocampal, right caudate, temporal, and middle cingulate areas. Whole-brain contrast (POSFW + NEGFW + NEUFW) - (POSPB + NEGPB + NEUPB) in blue shows overall brain activity level differences between 1st and 2nd halves of the experiment. Bilateral frontal, parietal, temporal and visual areas, not overlapping with emotion-related response areas in a) or in our main analyses, showed higher responses during FW than PB with no reverse effects. Statistical overlay threshold was set at $p < 0.001$, cluster-size > 10.

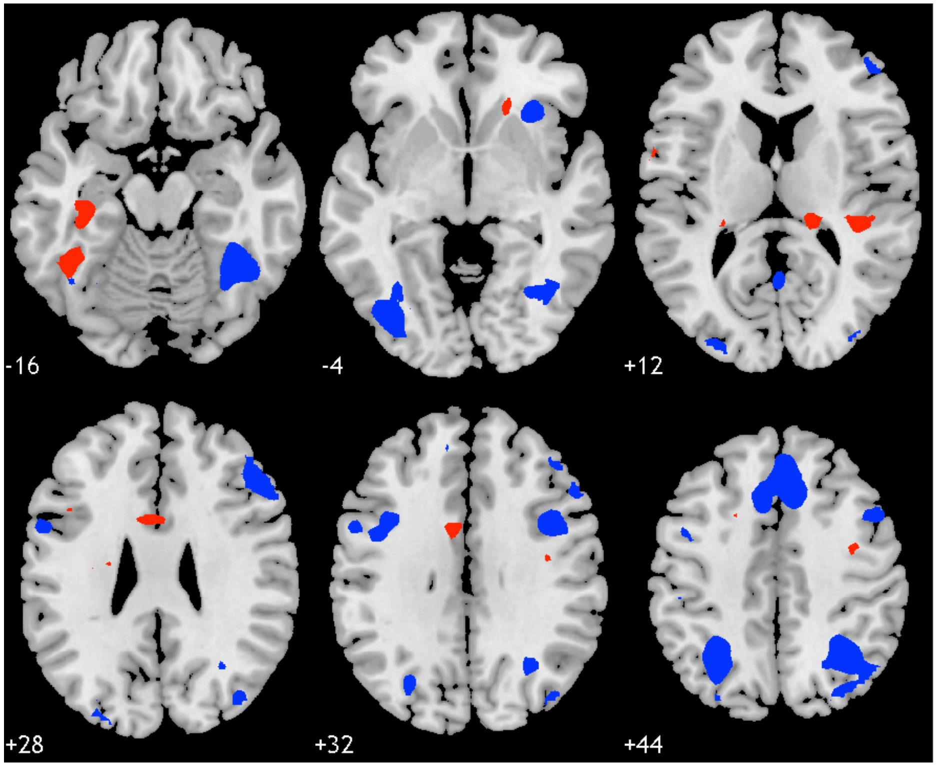

Supplement: Supplementary file 1 [file Data_Sheet_1.PDF]
